# Causal evidence for the role of the sensory visual cortex in visual short-term memory maintenance

**DOI:** 10.1098/rsos.230321

**Published:** 2023-04-19

**Authors:** Phivos Phylactou, Andria Shimi, Nikos Konstantinou

**Affiliations:** ^1^ Department of Rehabilitation Sciences, Faculty of Health Sciences, Cyprus University of Technology, Limassol 3041, Cyprus; ^2^ Department of Psychology, Faculty of Social Sciences and Education, University of Cyprus, CY-1678 Nicosia, Cyprus

**Keywords:** visual short-term memory, sensory visual cortex, working memory, sensory recruitment

## Abstract

The role of the sensory visual cortex during visual short-term memory (VSTM) remains controversial. This controversy is possibly due to methodological issues in previous attempts to investigate the effects of transcranial magnetic stimulation (TMS) on VSTM. The aim of this study was to use TMS, while covering previous methodological deficits. Sixty-four young adults were recruited to participate in two experiments (Experiment 1: *n* = 36; Experiment 2: *n* = 28) using a VSTM orientation change-detection task under TMS. Monocular vision was ensured using red-blue goggles combined with red-blue stimuli. Double-pulse TMS was delivered at different times (Experiment 1: 0, 200 or 1000 ms; Experiment 2: 200, 1000 ms) during a 2 s maintenance phase, on one side of the occipital hemisphere. In Experiment 2, a sham TMS condition was introduced. Decreased detection sensitivity (*d′*) in the ipsilateral occipital hemisphere to visual hemifield, and in the real TMS (compared with sham TMS) condition indicated inhibitory TMS effects, and thus, a causal involvement of the sensory visual cortex during early (200 ms) and late (1000 ms) maintenance in VSTM. These findings are aligned with sensory recruitment, which proposes that both perceptual and memory processes rely upon the same neural substrates in the sensory visual cortex. The methods used in this study were preregistered and had received in-principle acceptance on 6 June 2022 (Stage 1 protocol can be found in: https://doi.org/10.17605/OSF.IO/EMPDT).

## 1. Introduction

Visual short-term memory (VSTM) enables us to maintain in mind, for a short period of time, visual representations that are no longer present, in order to complete task-oriented goals. VSTM protects visual information against interference, making representations available for cognitive processing, and thus provides the essential link between perception and higher cognitive functions, underpinning our ability for complex thought and action [1]. For decades, cognitive scientists have studied the neural correlates of VSTM, establishing the role of specific brain areas such as the prefrontal and parietal areas in VSTM [2–14]. Although much research has attempted to understand the neural architecture of VSTM, it is still unclear if activity in the sensory visual cortex (area V1) is required for successfully maintaining visual information in short-term memory. Even though it is established that the sensory visual cortex is primarily engaged in encoding visual information [12,15–18], results are controversial with regard to its involvement in VSTM maintenance [3,12–15,18–24].

Traditionally, VSTM was investigated under the scope of sustained neural activity [25], suggesting that during VSTM tasks, neural activity potentials are maintained online in frontal and parietal cortical areas (e.g. [26,27]). However, more recently this traditional view has been challenged [11,28,29] following methodological advances in computational methods of neuroimaging data. Specifically, using multivariate analyses, it has been shown that VSTM contents can be decoded in the sensory visual area, in the absence of sustained brain activity [30,31]. In addition, early visual brain areas were shown to respond to specific visual features during VSTM maintenance, such as orientation [30–32], contrast [33] and direction of movement [34]. These findings led to the introduction of the sensory recruitment hypothesis, according to which the sensory visual cortex is an essential part of the brain network responsible for successfully maintaining information about elemental visual features in VSTM ([18,30,31,35–37]; for recent reviews see [20,23]). In summary, this evidence indicated that early visual areas (such as area V1) have a dual function: they are involved in the precise sensory encoding of elemental visual features (e.g. contrast, orientation, spatial frequency, direction of motion, speed of motion), and the short-term maintenance of this information.

Indeed, sensory visual cortex neurons are ideal candidates for short-term maintenance because they exhibit highly selective tuning for specific visual features. Utilizing specialized regions of the visual cortex to support VSTM might be a highly efficient way to avoid recoding remembered information in other distal networks. Moreover, the high degree of the sensory visual cortex selectivity is not observed in higher-order areas, whereas such selectivity is critical for remembering subtle distinctions between stimuli. Even though evidence using multivariate analyses has supported stimulus-specific activation in frontal and parietal areas [38,39], this view has recently been contended [40]. Yet, others have argued that storing information in sensory visual cortex leaves memory representations susceptible to overwriting as new stimuli are processed, and that networks in sensory areas are not sufficiently wired to support the type of recurrent activity thought to support VSTM [12–14]. Given that higher-order brain areas lack the visual selectivity of early sensory areas, it is still unclear how people can maintain specific visual features, such as the precise orientation of a visual stimulus, with minimal decay over some seconds [41].

The controversial evidence regarding sensory recruitment [3,12–15,18–23] has driven a debate in the current literature as to whether the sensory visual cortex is indeed involved in VSTM maintenance [42,43] or whether its role is restricted to the perception of visual information [12–14,44]. Specifically, Xu [12–14,44] argued that given the essential role of the sensory visual cortex during perception (see [15–19,29]), information maintenance by the sensory visual cortex leaves representations susceptible to overwriting as it processes new incoming stimuli. To reaffirm sensory recruitment, counterarguments (e.g. [42,43]) proposed that the sensory visual cortex protects representations by utilizing processes, such as between layer top-down signals in area V1 [45,46]. These processes were described as being similar to those employed by the prefrontal cortex during attention modulation (see [43]), when differentiating between mnemonic and perceptual information (e.g. [47]). Additionally, it has been proposed that the interaction between memory representations and perceptual input might be beneficial instead of detrimental to VSTM. For example, VSTM representations can improve perceptual continuity and goal-related behaviour by biasing perceptual input [48,49]. Further, Xu [12] pointed out that the sensory visual cortex is not sufficient to support sustained VSTM activity, and sustained activity recorded in the sensory visual cortex most likely relates to feedback from higher-order brain areas. However, alternative explanations proposed that sustained activity in the prefrontal cortex might echo a biasing signal to protect or direct attention toward goal related VSTM representations, rather than reflect VSTM representations *per se* [21,29,50–52].

To explore sensory recruitment, researchers have implemented many methodological approaches, such as functional magnetic resonance imaging (fMRI) and psychophysical experiments, but these have yielded mixed results (e.g. [2,53–55]). Several reasons have been proposed to explain these mixed results, such as activity silent mechanisms, feed-forward processes, lack of causal evidence, methodological differences and in some cases methodological oversights [12–15,18,20,23,29,56]. An ideal hypothetical scenario for investigating whether sensory visual cortex is required for VSTM maintenance would involve its complete inactivation during the retention interval of a VSTM task and reactivation at the onset of the memory probe display [42]. Such an experimental design could yield causal evidence as to whether the sensory visual cortex is a necessary component of the brain network responsible for the short-term maintenance of elemental visual features. Although such an experiment is impossible to be carried out, brain stimulation using transcranial magnetic stimulation (TMS) during the retention interval of a VSTM task can approximate this scenario. TMS uses a coil to transfer electromagnetic stimulation at localized brain areas. TMS targeted at the sensory visual cortex has been shown to directly interfere with cortical activity, making the exploration of causal evidence plausible [16,22,57].

Previous studies have attempted to investigate the role of the sensory visual cortex in VSTM using TMS, combined with delayed change-detection or match-to-sample tasks ([24,58–62]; for a review see [63]). In these tasks, a memory array (i.e. a set of stimuli that participants are asked to remember) is presented to participants, followed by a maintenance delay period. Subsequently, participants are requested to compare (or match) a probe with the earlier memory array. The sensory visual cortex is stimulated at different time points during the maintenance delay period, in order to make causal inferences based on the temporal point of the TMS interference. In most experiments, stimulation was induced on the sensory visual cortex of one hemisphere, while stimuli were presented either in the ipsilateral or contralateral (to the stimulation site) visual hemifield in a counterbalanced manner [24,58,60,62]. To draw evidence and reach a conclusion, comparisons between the ipsilateral versus the contralateral conditions [24,58,60,62] and between real versus sham TMS [58–61] were considered.

**Figure 1.**
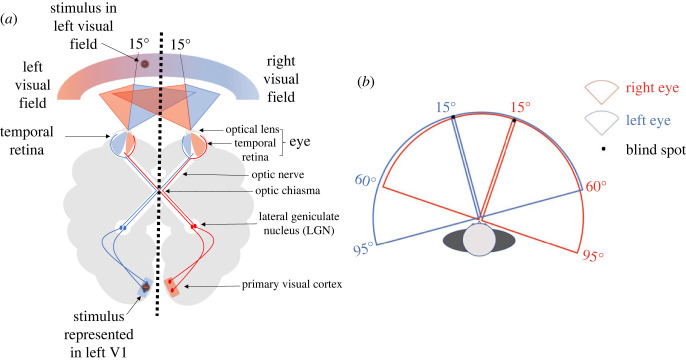
Neuroanatomy of the visual pathway. (*a*) In dichoptic presentation (represented by black vertical dotted line), a stimulus presented on the left visual field cannot be perceived by the right eye and it is therefore represented only in the ipsilateral V1 (i.e. left V1 in this example). (*b*) Visual field angle of the left and right eye. Stimuli presented within 15° of visual angle off fixation are perceived by both eyes.

As with different methodological approaches, results from previous TMS studies were mixed with regard to the sensory recruitment hypothesis. Specifically, some of the studies supported the sensory recruitment hypothesis [58,59,61], some rejected it [60,62], while others were unclear [64]. After a careful examination of the methods used in previous TMS studies, we suggest that the inconclusive findings are due to several important methodological issues that may have underestimated the contribution of the sensory visual cortex in VSTM. The most vital issue in the majority of these TMS studies is that previous researchers considered that, when information was presented on one side of the visual hemifield (either right or left side near the centre of the monitor), the information was processed by the contralateral sensory visual cortex. Therefore, stimuli were always presented binocularly to the participants either in the left or right visual field, and a contralateral sensory visual cortex TMS was applied and compared with an ipsilateral control condition (figure 1*a*). However, considering the neuroanatomy of the visual pathway system, the binocular presentation of stimuli either left or right close to the midline of the visual field—as was the case in the majority of the previous studies—does not accurately correspond to the contralateral sensory visual cortex, and could in fact be processed by the ipsilateral cortex if presented within 15° of visual angle from midline [65,66]. It is also possible that information enters the sensory visual cortex in both brain hemispheres [46,67] since the visual fields of both eyes overlap in certain areas (within 15° of visual angle, figure 1*b*; [66]). Consequently, some TMS effects can be falsely interpreted or remain undetectable (e.g. if information processing happens in both hemispheres despite the contralateral and ipsilateral conditions; [68]; see also [57]). For example, as pointed out in a recent review of the sensory visual cortex TMS VSTM literature [63], a study that considers the contralateral TMS condition as the experimental condition and the ipsilateral side as the control condition will interpret a performance drop (e.g. contralateral performance is less than ipsilateral performance) as an inhibitory TMS effect. Nevertheless, considering the evidence supporting the role of the ipsilateral sensory visual cortex in visual processing [46] and the neuroanatomy of the visual pathway [66], it is possible that the ipsilateral sensory visual cortex is in reality the experimental condition. As such, the conclusion of this study might turn out to be the opposite (e.g. facilitation effects, since ipsilateral accuracy is greater than contralateral accuracy), if the experimental and control conditions are inversely defined.

Another important shortcoming of the TMS literature relates to the complexity of the stimuli used in the memory array. In a given memory array, there is a minimal representational requirement for VSTM, based on the core features (e.g. colour, orientation, shape) of stimuli. A greater combination of stimuli features increases complexity and VSTM capacity requirements [69]. Previous TMS studies used various stimuli in their memory tasks, some of which were complex stimuli such as abstract shapes [64]. However, the evidence leading to the sensory recruitment hypothesis emphasized the selective engagement of the sensory visual cortex in elemental visual features such as orientation, contrast and direction of movement [30–33]. For example, Jia *et al.* [59], indeed found a strong TMS effect in a VSTM task requiring participants to remember the elemental visual feature of orientation of one grating. However, in a study requiring participants to remember either one (low load) or three (high load) abstract shapes (that are thought to be complex stimuli consisting of a combination of elemental visual features; [64]), TMS did not affect performance in the low-load condition of remembering a complex shape (TMS effects were evident only during the high-load condition). Such findings suggest that when stimulus complexity increases, higher-order brain areas, such as the intraparietal sulcus [70,71] and the posterior parietal cortex [72], might be more actively recruited for VSTM. Thus, the neural processes required for successful maintenance of complex visual stimuli in VSTM might be more dependent on higher-order brain areas than those required for simple stimuli consisting of elemental visual features, given the high selectivity of sensory visual cortex in processing of elemental features [23]. This might explain some of the null effects of sensory visual cortex TMS during the memory delay, since complex representations are probably protected through a more distributed VSTM network ([20]; see also [42,43]). Hence, it is possible that some of the previous studies failed to find evidence in favour of the sensory visual cortex involvement in VSTM due to using complex, rather than simple, stimuli.

Therefore, in order to provide causal evidence for the role of the sensory visual cortex during VSTM maintenance more reliably, the methodological limitations of previous TMS studies need to be addressed. In particular, the two visual hemifields must be reliably separated so that the visual input is processed by only one occipital hemisphere. One way to reliably separate the sensory visual cortex hemisphere that processes the information entering the visual field is to present the stimuli monocularly. To achieve monocular stimulus presentation, similar methodological principles as those used in binocular rivalry can be implemented [73]. In binocular rivalry, different images overlapping in the visual field are presented separately to each eye. Therefore, by presenting an image corresponding only to one eye (thus avoiding rivalry), stimuli will enter the sensory visual cortex monocularly [74]. Also, given the V1 neuronal response to specific visual features, the memory array should consist of an elemental visual feature known to selectively correspond to the sensory visual cortex, such as orientation [30–32,59,75].

Previous TMS studies stimulated the sensory visual cortex at various time points during VSTM maintenance, with variable results (e.g. [60,62,64]; for reviews see [12,63]). For example, Rademaker *et al*. [60] interfered with sensory visual cortex TMS at 0 and 900 ms into a 2 s delay period, after the offset of a memory array presented for 200 ms. Similarly, van Lamsweerde *et al*. [62] stimulated at 0, 100 and 200 ms during a 1 s delay period, which followed a 100 ms memory array. In another study, van de Ven *et al*. [64] induced TMS at 100, 200 and 400 ms of a 1.5 s delay period, after the presentation of a 150 ms memory array. Some studies indicated that TMS effects were stronger for earlier stimulation (up to 200 ms; [60,62]), compared with later stimulation at 400 [64] and 900 ms [60]; however, other studies indicated that TMS after 200 ms was stronger [64]. Based on a recent meta-analysis examining the effects of TMS on VSTM performance during the maintenance period, most studies differentiated between earlier (up to 200 ms into the maintenance period) and later (after 200 ms; usually halfway into the maintenance period) stimulation [63]. The meta-analysis provided evidence for a strong TMS effect (*g* = 0.8) during earlier TMS, and a moderate effect (*g* = 0.5) during later TMS; however, further analyses indicated that the TMS effects were not significantly different between the two timing conditions (overall effect *g =* 0.58). In the current work, we also differentiated between early and late TMS, by considering the outcomes of previous studies [60,62,64], and thus, to test our main question of whether the sensory visual cortex is involved in visual short-term memory, we examined the effects of TMS on behavioural performance separately for stimulation induced at 200 and 1000 ms (halfway) into the delay period. Further to our main hypotheses, exploratory analyses were performed in order to replicate and explore any similar findings concerning a different TMS effect size for earlier compared with later stimulation.

In short, the objective of the current study was to provide causal evidence for the role of the sensory visual cortex during early (200 ms) and/or late (1000 ms) VSTM maintenance using TMS, while ensuring monocular vision. In two experiments, stimuli were presented in the centre of the visual field, which were viewed monocularly. Therefore, based on the neuroanatomy of the visual pathway [65–67], it was expected that visual information will initially be processed solely by the ipsilateral (to the eye receiving the information) sensory visual cortex. As a result, and contrary to past experiments, the contralateral sensory visual cortex was the control condition. To explore our main question of whether the sensory visual cortex is involved in VSTM maintenance, our hypotheses focused on testing differences in detection sensitivity [76] for a VSTM task in two experiments. In Experiment 1, detection sensitivity was compared between the ipsilateral and contralateral conditions when stimuli were presented monocularly and TMS was applied (i) during perceptual processing (outcome neutral condition; 0 ms after stimulus onset; H1), (ii) during early information maintenance (200 ms after stimulus onset; H2), or (iii) during late information maintenance (1000 ms after stimulus onset; H3). More specifically, Experiment 1 enabled us to replicate previous, similar, TMS studies, at two different temporal points during the memory delay period, at an early (200 ms condition) and late (1000 ms) maintenance time point. Given the established role of the sensory visual cortex during perceptual processing (0 ms condition), the outcome neutral condition in Experiment 1 (ipsilateral versus contralateral *d*′ in 0 ms TMS condition; see H1 in table 1) was employed to evaluate the sufficiency of our methods to successfully manipulate sensory visual cortex activity with TMS. However, as discussed below, it is likely that a comparison between the ipsilateral and contralateral conditions alone is inadequate to explore the effects of TMS, for example, due to feedback and/or feedforward processes or due to TMS interference affecting both sensory visual cortex hemispheres (see *Experimental design*). Therefore, in a second experiment, further to the ipsilateral versus contralateral comparison (H4 and H6), we tested whether VSTM performance differed between a TMS and a sham TMS condition (i) during early information maintenance (200 ms after stimulus onset; H5) and (ii) during late information maintenance (1000 ms after stimulus onset; H7). Table 1 presents a detailed description of the main research hypotheses for each experimental condition.

**Table 1.** Design Table.

question	hypothesis	sampling plan	analysis plan	interpretation given to different outcomes
**Experiment 1**				
Q1: Is sensory visual cortex necessary during the perceptual processing of information in visual short-term memory?	H1: Given the established role of the sensory visual cortex during visual perception, we hypothesize that evidence for a difference between the ipsilateral and contralateral conditions will be present when sensory visual cortex TMS is induced at 0 ms (i.e. during memory sample presentation).	Sample updating with a stopping rule set at BF_10_ > 3 or <1/3 for all three paired *t*-tests.	Paired *t*-test between the ipsilateral and contralateral 0 ms condition on *d′* using a Cauchy prior centred around zero and width *r* = 0.58 (B_C(0,.58)_).	The 0 ms condition works as an outcome neutral test or positive control.
		Healthy individuals with normal or corrected to normal colour vision.	Simulation (*n* = 10 000) indicated that with a sample of 40 participants, given a true effect of *g* = 0.58, a BF_10_ > 3 is evident in 100% of the simulations, and, given a null effect (*g* = 0), a BF_10_ < 1/3 is evident in 70% of the simulations.	Evidence in support of the alternative hypothesis (i.e. TMS during the presentation of the memory sample affects VSTM performance) will indicate that our methods are reliable to detect a sensory visual cortex TMS effect between ipsilateral and contralateral stimulation. In such case, the sample mean distribution will indicate whether the effects of TMS are inhibitory (sample mean < 0) or facilitatory (sample mean > 0).
		To ensure counterbalancing a minimum of 20 participants will be recruited.		Given the well-established role of the sensory visual cortex in perception, evidence in support of the null hypothesis (i.e. VSTM performance not affected by sensory visual cortex stimulation during presentation of the memory sample) will indicate that the methods implemented might be insufficient to detect a TMS effect (e.g. possibly due to TMS affecting both hemispheres and/or due to feedforward mechanisms of the sensory visual cortex), which will be further explored with the addition of the sham TMS condition in Experiment 2.
		Due to time and resource constraints a maximum of 40 participants will be recruited.		If evidence is found in favour of the null hypothesis, but the alternative hypotheses are supported in H2 and/or H3, the results of Q1, Q2, and Q3 will be deemed inconclusive, probably due to false-negative (in H1) or false-positive (H2 and/or H3) errors.
Q2: Is sensory visual cortex necessary during the early maintenance of information in visual short-term memory?	H2: We hypothesize that evidence for a difference between the ipsilateral and contralateral conditions will be present when sensory visual cortex TMS is induced at 200 ms.		Paired *t*-test between the ipsilateral and contralateral 200 ms condition on *d′,* B_C(0,.8)_ prior.	The 200 ms TMS condition will provide evidence in support for or against the involvement of the sensory visual cortex during early information maintenance in VSTM. If evidence for the alternative hypothesis is found, the sample mean distribution will indicate whether the effects of TMS are inhibitory (sample mean < 0) or facilitatory (sample mean > 0).
			Simulation (*n* = 10 000) indicated that with a sample of 40 participants, given a true effect of *g* = 0.8, a BF_10_ > 3 is evident in 100% of the simulations, and, given a null effect (*g* = 0), a BF_10_ < 1/3 is evident in 80% of the simulations.	In the case that evidence in favour of the alternative hypothesis is found, but a failure of reproducing similar effects in H4, the results of Q2 and Q4 will be deemed inconclusive due to reproducibility failure.
				Evidence for the null hypothesis will indicate either that the sensory visual cortex is not involved in early VSTM maintenance or that ipsilateral versus contralateral comparisons are insufficient to detect a sensory visual cortex TMS effect. If evidence for a null hypothesis is found between the ipsilateral and contralateral condition but evidence for the alternative is found in H5 (see also *interpretation given to different* *outcomes* for Experiment 2), it indicates that TMS effects are undetectable between the ipsilateral and contralateral conditions with our methods (e.g. due to TMS spreading to both hemispheres and/or due to feedforward mechanisms). If evidence in favour of the null hypotheses is found for both H2 and H5, this will indicate that sensory visual cortex is not involved in early VSTM maintenance.
Q3: Is the sensory visual cortex necessary during the late maintenance of information in visual short-term memory?	H3: We hypothesize that evidence for a difference between the ipsilateral and contralateral conditions will be present when sensory visual cortex TMS is induced at 1000 ms.		Paired *t*-test between the ipsilateral and contralateral 1000 ms condition on *d′,* B_C(0,.5)_ prior.	The 1000 ms TMS condition will provide evidence in support for or against the involvement of the sensory visual cortex during late information maintenance in VSTM. If evidence for the alternative hypothesis is found, the sample mean distribution will indicate whether the effects of TMS are inhibitory (sample mean < 0) or facilitatory (sample mean > 0).
			Simulation (*n* = 10 000) indicated that with a sample of 40 participants, given a true effect of *g* = 0.5, a BF_10_ > 3 is evident in 100% of the simulations, and, given a null effect (*g* = 0), a BF_10_ < 1/3 is evident in 63% of the simulations.	In the case that evidence in favour of the alternative hypothesis is found, but a failure of reproducing similar effects in H6, the results of Q3 and Q5 will be deemed inconclusive due to reproducibility failure.
				Evidence for the null hypothesis will indicate either that the sensory visual cortex is not involved in late VSTM maintenance or that our methods of ipsilateral versus contralateral comparisons are insufficient to detect a sensory visual cortex TMS effect. If evidence for a null hypothesis is found between the ipsilateral and contralateral condition but evidence for the alternative is found in H7 (see also *interpretation given to different* *outcomes* for Experiment 2), it indicates that TMS effects are undetectable between the ipsilateral and contralateral conditions with our methods. If evidence in favour of the null hypotheses is found for both H3 and H7, it indicates that sensory visual cortex is not involved in late VSTM maintenance.
**Experiment 2**				
Q4: Is the sensory visual cortex necessary during the early maintenance of information in visual short-term memory?	H4: We aim to replicate the effects of Exeriment 1 (H2) for the difference between the ipsilateral and contralateral conditions when sensory visual cortex TMS is induced at 200 ms.	Sample updating with a stopping rule set at BF_10_ > 3 or < 1/3 for all four paired *t*-tests.	Paired *t*-test between the ipsilateral and contralateral 200 ms condition on *d′,* B_C(0,.8)_ prior.	Evidence for the alternative hypothesis indicates an involvement of the sensory visual cortex in early VSTM maintenance, with the sample mean distribution indicating whether the effects of TMS are inhibitory (sample mean < 0) or facilitatory (sample mean > 0).
		Healthy individuals with normal or corrected to normal colour vision.	Simulation (*n* = 10 000) indicated that with a sample of 40 participants, given a true effect of *g* = 0.8, a BF_10_ > 3 is evident in 100% of the simulations, and, given a null effect (*g* = 0), a BF_10_ < 1/3 is evident in 80% of the simulations.	Evidence for the null hypothesis will indicate either that the sensory visual cortex is not involved in early VSTM maintenance or that our methods of ipsilateral versus contralateral comparisons are insufficient to detect a sensory visual cortex TMS effect. However, if evidence for a null hypothesis is found between the ipsilateral and contralateral condition but evidence for the alternative is found in H5, then evidence in favour of the null hypothesis indicates that TMS effects are undetectable between the ipsilateral and contralateral conditions. If evidence in favour of the null hypotheses is found for H2, H4 and H5, it indicates that sensory visual cortex is not involved in early VSTM maintenance.
		To ensure counterbalancing a minimum of 20 participants will be recruited.		
		Due to time and resource constraints a maximum of 40 participants will be recruited.		
	H5: We hypothesize that evidence for a difference between the real and sham TMS conditions will be present when sensory visual cortex TMS is induced at 200 ms.		Paired *t*-test between the real and sham 200 ms TMS condition on *d′* across hemispheres*,* B_C(0,.8)_ prior.	Evidence for the alternative hypothesis indicates an involvement of the sensory visual cortex in early VSTM maintenance.
			Simulation (*n* = 10 000) indicated that with a sample of 40 participants, given a true effect of *g* = 0.8, a BF_10_ > 3 is evident in 100% of the simulations, and, given a null effect (*g* = 0), a BF_10_ < 1/3 is evident in 80% of the simulations.	If evidence is found between real and sham sensory visual cortex TMS across hemispheres, but no evidence is found between the ipsilateral and contralateral conditions (H2, H4), then hemisphere comparisons alone were insufficient to detect the TMS effect.
				The sample mean distribution indicating whether the effects of TMS are inhibitory (sample mean < 0) or facilitatory (sample mean > 0).
				Evidence for the null hypothesis will indicate that the sensory visual cortex is not involved in early VSTM maintenance.
				If evidence in favour of the null hypothesis is found, but the alternative hypothesis is supported in H2 and/or H4, the results for Q4 will be deemed inconclusive due to a failure of replication.
Q5: Is the sensory visual cortex necessary during the late maintenance of information in visual short-term memory?	H6: We aim to replicate the effects of Experiment 1 for the difference between the ipsilateral and contralateral conditions when sensory visual cortex TMS is induced at 1000 ms.		Paired *t*-test between the ipsilateral and contralateral 1000 ms condition on *d′,* B_C(0,.5)_ prior.	Evidence for the alternative hypothesis will indicate an involvement of the sensory visual cortex in late VSTM maintenance, with the sample mean distribution indicating whether the effects of TMS are inhibitory (sample mean < 0) or facilitatory (sample mean > 0).
			Simulation (*n* = 10 000) indicated that with a sample of 40 participants, given a true effect of *g* = 0.5, a BF_10_ > 3 is evident in 100% of the simulations, and, given a null effect (*g* = 0), a BF_10_ < 1/3 is evident in 63% of the simulations.	Evidence for the null hypothesis will indicate either that the sensory visual cortex is not involved in late VSTM maintenance or that our methods of ipsilateral versus contralateral comparisons are insufficient to detect a sensory visual cortex TMS effect. If evidence for a null hypothesis is found between the ipsilateral and contralateral condition but evidence for the alternative is found in H7, then evidence in favour of the null hypothesis indicates that TMS effects are undetectable between the ipsilateral and contralateral conditions with our methods. If evidence in favour of the null hypotheses is found for H3, H6 and H7, it will indicate that the sensory visual cortex is not involved in early VSTM maintenance.
	H7: We hypothesize that evidence for a difference between the real and sham TMS conditions will be present when sensory visual cortex TMS is induced at 1000 ms.		Paired *t*-test between the real and sham 1000 ms condition on *d′* across hemispheres*,* B_C(0,.5)_ prior.	Evidence for the alternative hypothesis will indicate an involvement of the sensory visual cortex in late VSTM maintenance.
			Simulation (*n* = 10 000) indicated that with a sample of 40 participants, given a true effect of *g* = 0.5, a BF_10_ > 3 is evident in 100% of the simulations, and, given a null effect (*g* = 0), a BF_10_ < 1/3 is evident in 63% of the simulations.	If evidence is found between real and sham TMS across hemispheres, but no evidence is found between the ipsilateral and contralateral conditions (H3, H6), it will indicate that hemisphere comparisons alone were insufficient to detect the TMS effect.
				The sample mean distribution indicating whether the effects of TMS are inhibitory (sample mean < 0) or facilitatory (sample mean > 0).
				Evidence for the null hypothesis will indicate that the sensory visual cortex is not involved in late VSTM maintenance.
				If evidence in favour of the null hypothesis is found, but the alternative hypothesis is supported in H3 and/or H6, the results for Q5 will be deemed inconclusive due to a failure of replication.

## 2. Methods

The hypotheses and methods of this study were preregistered and have received in-principle acceptance on 6 June 2022 after undergoing peer review. The accepted Stage 1 protocol can be accessed at https://doi.org/10.17605/OSF.IO/EMPDT [77].

### 2.1. Design

#### 2.1.1. Apparatus and stimuli

**Figure 2.**
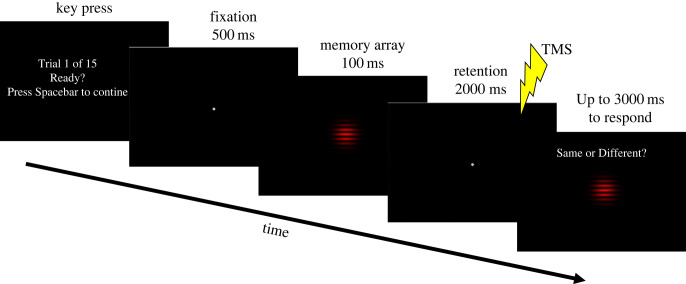
Stimuli and experimental procedure. An example of the delayed change-detection task used in Experiments 1 and 2. The trial begins with a screen indicating the trial number, requesting a key press to proceed. This is followed by a 500 ms fixation dot. Next, the memory array, consisting of either a red or blue Gabor patch, is shown for 100 ms and participants are asked to memorize its orientation. From the memory array onset, a 2000 ms retention period is presented. During the retention phase, double-pulse TMS is induced at either the left or right sensory visual cortex. In Experiment 1, stimulation is induced at 0, 200 or 1000 ms after the memory array onset. In Experiment 2, either real or sham stimulation is induced at 200 or 1000 ms after the memory array onset. Following the retention period, a probe stimulus is presented at the centre of the screen for up to 3000 ms (or until a response is given), where participants have to respond whether it matches the remembered stimulus or not.

A Magstim Super Rapid^2^ (MagStim, Whitland, Wales, UK SA34 0HR) stimulator was used for inducing TMS. A Magstim D70 Alpha Flat Coil (Uncoated) delivered a double-pulse TMS at the different experimental conditions, while a sham coil was used to control for noise and other TMS artefacts (in Experiment 2). The sham coil looks identical to the D70 Alpha Flat Coil, but it is equipped with thicker shield, restricting it from inducing magnetic fields that interfere with brain activity. The double-pulse TMS was induced with a frequency of 10 Hz, meaning that stimulation was delivered by two pulses separated by a duration of 100 ms. A 10 Hz double-pulse TMS was chosen to ensure the reliability of the outcome neutral condition. Specifically, the first pulse was induced at the beginning of stimulus presentation and the second pulse at stimulus offset (see below). Given the possibility that a long encoding time (approx. 100 ms) can lead to successful consolidation despite masking interference [78–80], the double-pulse TMS ensured that interference with regular brain activity is introduced throughout the consolidation process [78,79]. For comparison and consistency reasons, the double-pulse TMS was used in all experimental conditions. The stimuli and all experimental procedures were designed and controlled using Python and PsychoPy [81], which were run on an HP PRODESK desktop computer. To control the TMS, the MagPy TMS package was used [82]. Stimuli were presented on a 21.5″ Philips 226V^la^ monitor with a 60 Hz refresh rate. A chinrest was placed to ensure that participants maintained a viewing distance of 57 cm from the monitor. Stimuli consisted of either a red (RGB: 255, 0, 0) or a blue (RGB: 0, 0, 255) Gabor patch, which was oriented either horizontally or with a clockwise or counter-clockwise tilt from the horizontal axis, presented on a black (RGB: 0, 0, 0) background (figure 2). The Gabor patch consisted of a Gaussian envelope with a standard deviation of 0.39° (in degrees of visual angle), 0.001° frequency and had a 1° diameter. Stimuli were presented at fixation. To ensure that the memory array stimulus was viewed monocularly, stimuli were viewed through red/blue anaglyph goggles, consistent with previous research [83], where red stimuli were only viewed by the left eye and blue stimuli only by the right eye [73].

#### 2.1.2. Experimental design

Two experiments using the same delayed change-detection task were carried out. Participants were asked to compare the orientation of a probe with the orientation of a remembered grating (memory array) after a 2 s delay period (figure 2). In half the trials, the probe had the same orientation as the memory array. In the other half, the probe was oriented clockwise (25% of the trials) or counter-clockwise (25% of the trials) to the remembered grating (figure 2).

Experiment 1 was designed to allow for within-subject comparisons between the ipsilateral and contralateral stimulation conditions at three different TMS timing conditions. Timing conditions refer to the temporal distance of the stimulation after the memory grating's onset. The 0 ms timing condition worked as an outcome neutral test measurement to confirm that our method was reliable to detect TMS effects. Specifically, the first TMS pulse was induced at the onset of the stimulus (at 0 ms) and the second TMS pulse at the offset of the stimulus (at 100 ms, given that the two TMS pulses are separated by a duration of 100 ms). Thus, given the established role of the sensory visual cortex during visual perception [12,15–18], a significant difference in VSTM performance was expected in the ipsilateral compared with the contralateral condition (either facilitation or inhibition; for details, see table 1) in the 0 ms condition. The second, 200 ms, condition (first TMS pulse at 200 ms after stimulus onset and second TMS pulse at 300 ms after stimulus onset) shed light on the role of the sensory visual cortex during the early maintenance phase of VSTM, while the third, 1000 ms, condition (first TMS pulse at 1000 ms after stimulus onset and second TMS pulse at 1100 ms after stimulus onset) allowed the exploration of its role during the later maintenance period. These conditions lead to a two (ipsilateral/contralateral) by three (0/200/1000 ms) design. A total of 432 trials^1^ (144 trials per timing condition; 72 with ipsilateral TMS and 72 with contralateral TMS in each timing condition) were gathered, which were divided into six blocks of 72 trials each, and presented in a counterbalanced manner across participants.

Experiment 2 aimed to replicate the effects that were obtained in Experiment 1, while controlling for other factors that may cause or hinder our experimental effects, by adding a sham TMS control condition. In addition to controlling for TMS noise and other artefacts, a sham TMS control is important for three reasons. First, TMS interference may affect both hemispheres due to the visual input being processed by both hemispheres and thus any actual effects remain undetected [57,68]. Since Experiment 1 compared an ipsilateral with a contralateral condition, where stimulation is always present, it is plausible that TMS noise interferes in such a way, that an effect in behaviour is always present. Thus, if the additional noise by TMS affects the baseline condition, then comparisons between the ipsilateral and contralateral stimulation condition might not indicate any significant difference. By introducing a sham TMS condition, Experiment 2 controlled for this possibility, allowing comparisons between real and sham stimulation. Second, it is likely that the sensory visual cortex processes information in both hemispheres (e.g. due to feedforward and feedback mechanisms; [46,84]) so that stimulating only one of the two hemispheres is not enough to affect behavioural measures. Lastly, contrary to previous research, we suggested that visual information was initially processed by the ipsilateral sensory visual cortex when the stimulus was presented within approximately 15° of visual angle from midline [65–67]. However, without a sham control condition, it would be impossible to correctly interpret the direction of any possible effect. Specifically, in previous experiments, TMS was shown to either facilitate [58] or hinder [59,64] performance. It should be pointed out that the interpretations of such effects are unavoidably biased by the hypotheses. For example, if an effect is expected in the contralateral site, an increased performance might be interpreted as a facilitation effect but might, in reality, be due to hindering effects in the ipsilateral condition. Thus, given the neural basis of the visual pathway [65–67], along with the possible feedforward and feedback mechanisms of the sensory visual cortex (e.g. [45,85]; see also [84]), this is an important factor that must be controlled for. Therefore, Experiment 2, allowed comparisons between actual and sham stimulation on behaviour. Since sham TMS was introduced in Experiment 2, which worked as a baseline measurement, the 0 ms condition that was used as an outcome neutral condition in Experiment 1 was dropped. Therefore, in Experiment 2, only two timing conditions were used, at 200 ms (first TMS pulse at 200 ms after stimulus onset and second TMS pulse at 300 ms after stimulus onset) and 1000 ms (first TMS pulse at 1000 ms after stimulus onset and second TMS pulse at 1100 ms after stimulus onset), corresponding to an early maintenance phase and a late maintenance phase of VSTM, respectively. As in Experiment 1, the timing conditions refer to the temporal distance between stimulation and memory array onset. This led to a within-subject design, comparing differences between the ipsilateral and contralateral conditions, at two different TMS timing conditions, and two different stimulation conditions. These conditions created a two (ipsilateral/contralateral) by two (200/1000 ms) by two (TMS/sham TMS) design. In total, 576 trials (288 TMS conditions; 144 per timing condition, out of which 72 ipsilaterally and 72 contralaterally and 288 sham TMS conditions; 144 per timing condition 72 ipsilaterally and 72 contralaterally) were collected, which were divided into eight blocks of 72 trials and presented across participants in a counterbalanced fashion.

#### 2.1.3. Procedure

*Sensory visual cortex stimulation.* Before the main experiment, we localized the right or left sensory visual cortex of each participant [58,61,64] using the functional method of eliciting phosphenes [86] and the localization was counterbalanced across participants. Specifically, a tight cap was placed on each participant's head and the inion was marked. Participants were blindfolded but instructed to keep their eyes open using a hollow blindfold. The coil was placed 2 cm above the inion and 1 cm laterally (either left or right, based on the participant's group). Starting at a 60% TMS output power, a single-pulse TMS was delivered and participants orally reported whether they have seen phosphenes or not (by saying out loud ‘yes’ or ‘no’). If no phosphenes are reported after three consecutive stimulations, the procedure was repeated by moving the coil in a 1 × 1 cm grid around the initial stimulation point by approximately 0.2 cm, inducing three single-pulse TMS at each position. If a participant still failed to report phosphenes, the same procedure was repeated with a 5% increase on the stimulator output until phosphenes were reported, or until an 80% power on the stimulator was reached. If participants failed to report phosphenes, the localization procedure was repeated on the opposite cortex and if they still failed to perceive phosphenes, a fixed output set at 65% of the stimulator's maximum output was used, as has been done previously [58,87,88]. When the participants successfully reported phosphenes, a mark was placed on the cap and a mechanical arm stabilized the TMS coil, and, together with the chinrest, this held the participant's head stable on that point. The TMS coil was stabilized at the position where participants reported phosphenes as close to the centre of the visual field as possible, thus overlapping with stimulus presentation. Three additional single pulses were induced to confirm that participants experienced phosphenes, and thus the coil was placed correctly. Halfway through the experiments (after three blocks in Experiment 1, and after four blocks in Experiment 2), participants were blindfolded again, and three single pulses were induced on the mark, to confirm the induction of phosphenes and consequently stable coil placement. During this process, and if necessary, phosphene localization was repeated to adjust for possible drifts.

After localizing the sensory visual cortex, we estimated each participant's individual threshold by determining the required stimulation power output for perceiving phosphenes using an adjusted staircase method [89]. With the use of custom code, double-pulse TMS stimulation was induced on the localized sensory visual cortex at different stimulation output powers, and participants responded whether they have seen phosphenes or not via button press. Given their responses, the power decreased (if they reported phosphenes twice on a specific TMS power output consecutively) or increased (every time they failed to report phosphenes). Calculations based on the mean of the intervals where the power output changes direction (i.e. from higher power to lower or vice versa) produced an approximation of the stimulation power required to elicit phosphenes 50% of the time the sensory visual cortex was stimulated. Because this procedure was done with a blindfold over participants' eyes, stimulation power in the main experiments was set at 110% of the estimated threshold stimulation power to adjust for visual exposure that can affect the phosphene threshold [90].

To account for individual differences and avoid ceiling or floor effects in task performance, additional procedures were conducted before the main experiments. Specifically, the task was adjusted to each participant's perceptual ability to discriminate between orientation changes. A custom staircase procedure was implemented, where participants had to report whether a grating had a clockwise or counter-clockwise tilt from the horizontal axis. According to each participant's responses, the degrees of this tilt either decreased (when three consecutive correct responses were given) or increased (when a response was incorrect). An approximation of accurately discriminating the orientation difference 75% of the time was obtained by calculating the mean of the intervals where degree differences changed direction (i.e. from an increase in degrees to a decrease and vice versa). The gratings used in this staircase were identical to the experimental stimuli, and so this procedure was carried out twice, separately for the blue and red stimuli. For the main experiment, the orientation thresholds both for the red and blue stimuli were increased by 20%, to account for the increased cognitive demands of the main task. Furthermore, before the two main experiments, participants carried out a practice block, based on the results of the orientation discrimination staircase procedure (i.e. individual perceptual ability to discriminate orientation changes) of 24 trials without TMS stimulation to familiarize themselves with the experimental procedure. If accuracy in the practice block was less than 75%, the orientation discrimination staircase and practice block were repeated until the participant reached at least 75% accuracy. Participants were replaced if after four practice blocks their accuracy remained below 75%.

*Experiment 1.* Each trial began with a screen indicating the trial number for each block. To proceed to the next trial, participants needed to press the ‘spacebar’ key on the keyboard. Next, a 500 ms white fixation dot appeared on the centre of a black background, followed by the memory grating for 100 ms. The stimulus grating either had a horizontal orientation (50% of trials), a clockwise (25% of trials) or counter-clockwise (25% of trials) tilt. The tilt angle was fixed across all trials for each participant at the level determined using the staircase procedure described above. From stimulus onset, a 2000 ms delay period indicated by a centred fixation dot followed. Double-pulse TMS were pseudorandomly delivered at one of three different timing conditions after the memory onset; either 0, 200 or 1000 ms. At the end of the delay period, a probe stimulus appeared. In half of the trials, the probe was the same as the memory array stimulus. In the remaining 50% of trials, the probe was different as follows: if the memory array was horizontal, the probe was tilted clockwise (25% of the different-condition trials) or counter-clockwise (25% of the different-condition trials). If the memory array stimulus was tilted, then the probe was horizontal (50% of the different-condition trials). Participants had up to 3000 ms starting at probe onset to respond by placing their index and middle fingers on the arrow keys on the keyboard, indicating whether the orientation of the probe was the same (index finger; ‘left arrow key’) or different (middle finger; ‘down arrow key’) compared with the memory array grating. Feedback was provided only in the cases of no response or an incorrect response, by presenting the word ‘Wrong!’ in red letters in the centre of the screen for 1000 ms.

*Experiment 2.* The second experiment used the same delayed change-detection VSTM task as in Experiment 1. The difference in Experiment 2 is the introduction of a sham coil that delivered sham stimulation. TMS and sham TMS conditions were blocked in a counterbalanced order. In addition, given the sham TMS condition, the 0 ms condition of Experiment 1 that acted as an outcome neutral test, was dropped. At the end of Experiment 2, participants self-reported whether they noticed any differences between sham TMS and TMS.

### 2.2. Sampling plan

Healthy undergraduate and graduate students from the Cyprus University of Technology were recruited to participate voluntarily. Only individuals with normal or corrected to normal vision were included in the study. Prior to participation, participants were screened for colour deficiencies using the 10-item screening edition Ishihara colour deficiency test, and any individual who showed signs of colour blindness were excluded from the study.

For Experiment 1, sample updating with a stopping rule was set to *BF*_10_ > 3 or < 1/3 for all three paired *t*-tests that were performed. However, due to counterbalancing, a minimum of 20 participants (to ensure counterbalancing) or a maximum of 40 participants were to be recruited, given time and resource constraints. Specifically, after data collection for the first 20 participants was completed, we performed our analyses to check if the stopping rule was fulfilled. If any of the three *BFs* did not reach the stopping rule of >3 or <1/3, we continued with data collection, as follows: we recruited four additional participants and performed the analyses again. This process was to be repeated until all three *BFs* fulfilled the stopping rule, or until the maximum of 40 participants was reached. A similar sample updating process with a stopping rule (*BF*_10_ > 3 or < 1/3) was set for all four paired *t*-tests of Experiment 2. Similar to Experiment 1, a minimum of 20 participants (to ensure counterbalancing) or a maximum of 40 participants (due to constraints) were to be recruited for Experiment 2. Therefore, the total number of participants for both experiments was expected to range between 40 and 80 participants.

In order to confirm the adequacy of our proposed sample size, we simulated each of our registered *t-*tests 10 000 times. The simulation results indicated that for the outcome neutral condition a *BF*_10_ > 3 or *BF*_10_ < 1/3 was evident in 85% of the simulations. Specifically, assuming the alternative hypothesis is true for the outcome neutral condition with an expected effect size *g* = 0.58, a *BF* > 3 (median *BF*_10_ = 12.2 × 10^6^) was generated in 100% of the simulations, while assuming the null hypothesis is true (*g* = 0), a *BF*_10_ < 1/3 (median *BF*_10_ = 0.252) was produced in 70% of the simulations. For the encoding condition a *BF*_10_ > 3 or *BF*_10_ < 1/3 was evident in 90% of the simulation. In detail, assuming the alternative hypothesis is true in the encoding condition (*g* = 0.8), the simulation yielded a *BF*_10_ > 3 (median *BF*_10_ = 19.1 × 10^7^) in 100% of the simulations, and assuming the null hypothesis is true (*g* = 0), a *BF*_10_ < 1/3 (median *BF*_10_ = 0.189) was evident in 80% of the simulations. Lastly a *BF*_10_ > 3 or *BF*_10_ < 1/3 was evident in 81% of the simulations for the maintenance condition, where 100% of the simulations yielded a *BF*_10_ > 3 (median *BF*_10_ = 23.3 × 10^6^), assuming the alternative hypothesis is true (*g* = 0.5), and 63% of the simulations yielded a *BF*_10_ < 1/3 (median *BF*_10_ = 0.285), assuming the null hypothesis (*g =* 0) is true. The results of these simulations are consistent with previous work suggesting that a total of 40 participants is adequate to provide a *BF*_10_ > 3 or *BF*_10_ < 1/3 with a proportion of at least 80% [91].

### 2.3. Participants

#### 2.3.1. Experiment 1

**Figure 3.**
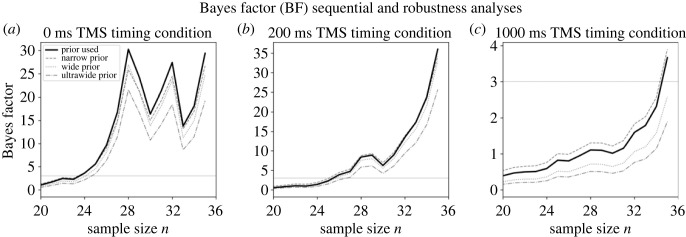
Sequential and prior robustness analysis for Experiment 1. Bayes factor (*BF*) sequential and robustness analysis for the (*a*) 0 ms, (*b*) 200 ms and (*c*) 1000 ms TMS timing conditions. Following our stopping rule, data collection in Experiment 1 stopped at 36 participants, when the predefined *BF* threshold (*BF* > 3; presented here as the grey horizontal solid line) was reached for all three registered analyses. The *BF* sequential analysis for each registered analysis is shown with a black solid line. The *BF* was informed by a Cauchy distribution centred on 0 with a scaling factor set to (*a*) *r =* 0.58, (*b*) *r =* 0.8 and (*c*) *r* = 0.5. To test the robustness of the *BF,* analyses were repeated for a narrow prior with *r* = 0.3 (dashed grey line), a wide prior with *r* = 1 (dotted grey line) and an ultrawide prior with *r* = 1.5 (dash-dotted grey line). TMS: transcranial magnetic stimulation.

Following the sequential procedure described above, data collection for Experiment 1 was stopped after collecting data from 36 (26 females) participants (figure 3) with mean age 24.25 (s.d. = 4.87) years. In total, 43 participants were recruited for Experiment 1; however, as per our preregistered sampling plan four participants were replaced due to VSTM task performance that was close to chance levels (accuracy less than 60%) and three participants were replaced due to vision deficiencies, which were self-reported during study debriefing (amblyopia for two participants, uncorrected astigmatism for one participant). All participants that were replaced were excluded from all analyses.

#### 2.3.2. Experiment 2

**Figure 4.**
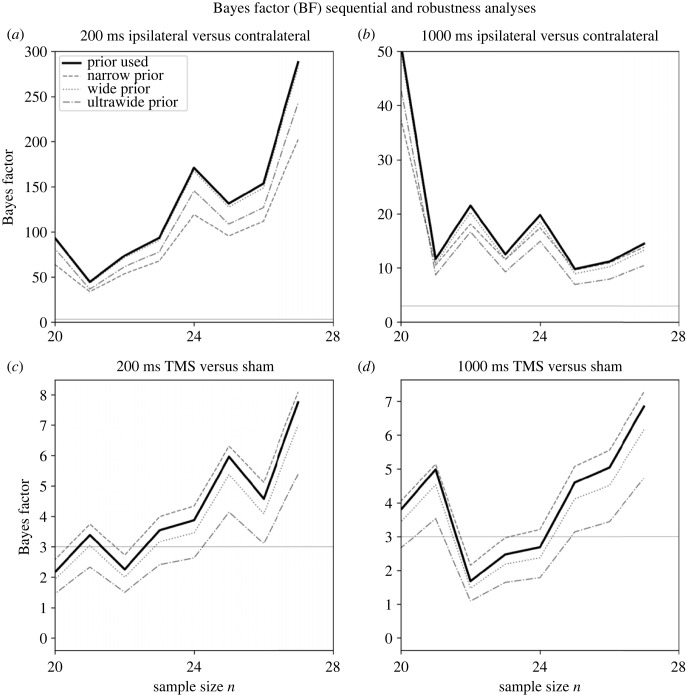
Sequential and prior robustness analysis for Experiment 2. Bayes Factor (*BF*) sequential and robustness analysis for TMS site (ipsilateral versus contralateral) in the (*a*) 200 ms and (*b*) 1000 ms timing conditions, and for the TMS condition (real versus sham) in the (*c*) 200 ms and (*d*) 1000 ms timing conditions. Following our stopping rule, data collection in Experiment 2 stopped at 28 participants, when the predefined *BF* threshold (*BF* > 3; presented here as the grey horizontal solid line) was reached for all three registered analyses. The *BF* sequential analysis for each registered analysis is shown with a black solid line. The *BF* was informed by a Cauchy distribution centred on 0 with a scaling factor set to (*a*, *c*) *r =* 0.8 and (*b*, *d*) *r =* 0.5. To test the robustness of the *BF,* analyses were repeated for a narrow prior with *r* = 0.3 (dashed grey line), a wide prior with *r* = 1 (dotted grey line), and an ultrawide prior with *r* = 1.5 (dash-dotted grey line). TMS: transcranial magnetic stimulation.

In Experiment 2 data collection was stopped, according to our sequential procedure, after collecting data from 28 (24 females) participants (figure 4) with mean age 20.29 (s.d. = 3.14) years. Thirty-two participants were recruited in total for Experiment 2; however, as per our preregistered sampling plan, three participants were replaced because of poor VSTM task performance (accuracy less than 60%) and one participant was replaced due to self-reported history of amblyopia during study debriefing. The results from the participants that were replaced were not included in any of the analyses.

### 2.4. Analysis plan

Analyses were conducted using Jamovi ([92], v. 2.3.13; https://www.jamovi.org), an openly available R-based statistical software.

#### 2.4.1. Experiment 1

The TMS site (ipsilateral versus contralateral) was the independent variable in Experiment 1. Since monocular vision was ensured, the ipsilateral condition refers to the situation where the TMS localized site (for example, right sensory visual cortex) was on the same side as the eye processing the stimulus (for example, right eye, and consequently the blue stimulus). The contralateral condition corresponds to when the TMS localized site (for example, right sensory visual cortex) did not match the side of the eye processing the stimuli (for example, left eye, and consequently red stimulus).

The main dependent variable that was considered is *d*′. The *d*′ variable is a signal detection theory indicator of detection sensitivity calculated by subtracting the standardized false alarm rate of responses from the standardized hit rate

$$d^{\prime }\;=\;z(H)\;\text{–}\;z(FA),$$

where *H* is the hit rate (i.e. correct responses of the probe being the same as the memory array grating) and *FA* is the false alarm rate (i.e. incorrect responses of the probe being the same as the memory array grating). These rates correspond to probabilities on the normal distribution, therefore *z(H)* and *z(FA)* are the *z-*scores that correspond to the normal distribution's tail *p*-values represented by *H* and *FA*.

In Experiment 1, we performed three Bayesian paired *t-*tests to calculate a Bayes factor; one *t*-test on TMS stimulation site (ipsilateral *d*′ versus contralateral *d*′) for each of the three TMS timing conditions (0, 200 and 1000 ms). Each *t*-test examined if the difference between the ipsilateral *d*′ and contralateral *d*′ differs from zero. The Bayes factor indicated the likelihood ratio of each alternative hypothesis over the null hypothesis (*BF*_10_), thus providing evidence for the likelihood of both hypotheses (table 1). The 0 ms timing condition worked as an outcome neutral test or positive control condition, in order to test our methods. Given that the effect of TMS might affect both hemispheres and/or that the sensory visual cortex processes information in both hemispheres through feedforward and feedback processes, it is possible that TMS effects between hemispheres remained undetected with our proposed methods. This possibility was tested in Experiment 2, with the introduction of sham TMS condition and statistical tests between real versus sham TMS across hemispheres. The 200 and 1000 ms timing conditions tested whether the sensory visual cortex is involved during early and late maintenance of visual information, respectively.

Each prior for the paired *t-*tests was described by a Cauchy distribution centred around zero (see [93]). Each prior was based on the results of a recent meta-analysis on the topic [63], which reported the standardized differences (Hedge's *g*) of accuracies and signal detection estimates between sensory visual cortex TMS and control conditions. These standardized differences were used to inform the width parameter of each Cauchy prior. In detail, by considering the overall effect size (*g =* 0.58), the effect size for early TMS (up to 200 ms; *g =* 0.80) and the effect size for late TMS (after 200 ms; *g =* 0.50) from our previous meta-analytic work [63], the width parameter of the Cauchy distribution corresponded to 0.58 for the 0 ms condition, to 0.8 for the 200 ms condition and to 0.5 for the 1000 ms condition, respectively.

#### 2.4.2. Experiment 2

In Experiment 2, the independent variables were the stimulation site (ipsilateral, contralateral) and the TMS condition (real, sham). As in Experiment 1, the dependent variable was the estimated detection sensitivity as measured with *d′*. Thus, for Experiment 2 we performed four paired *t*-tests; one *t*-test between ipsilateral *d′* versus contralateral *d′* for each of the two TMS timing conditions (200 and 1000 ms) only for the real TMS condition, and one paired *t*-test between real TMS *d′* versus sham TMS *d′* for each of the TMS timing conditions (200 and 1000 ms) across hemispheres. The stimulation site (ipsilateral versus contralateral) *t*-test was performed to replicate the results of Experiment 1 regarding the involvement of the sensory visual cortex during early (200 ms condition paired *t*-test) and late (1000 ms condition paired *t*-test) VSTM maintenance, by testing if the difference between ipsilateral *d′* and contralateral *d′* equalled to 0 (null hypothesis) or not (alternative hypothesis). The real TMS *d′* versus sham TMS *d′* comparison tested the effects of stimulation across hemispheres to provide evidence for the involvement of the sensory visual cortex during early (200 ms condition paired *t*-test) and late (1000 ms condition paired *t*-test) VSTM maintenance, by testing if the difference between real TMS *d′* and sham TMS *d′* equalled to 0 (null hypothesis) or not (alternative hypothesis). Further, it indicated whether the analyses between the stimulation site (ipsilateral versus contralateral) were insufficient to detect a TMS effect (e.g. if evidence was found in favour of the null hypotheses for ipsilateral versus contralateral tests and evidence for an alternative hypothesis was found in the real TMS versus sham TMS tests), or if the sensory visual cortex is not involved during early and/or late VSTM maintenance (evidence in favour of the null hypotheses in both ipsilateral versus contralateral and real versus sham TMS tests).

The priors which were used for the paired *t*-tests were described as a Cauchy distribution centred around 0 with a width set to 0.8 for the 200 ms condition and 0.5 for the 1000 ms condition, as estimated by the results of recent meta-analytic evidence [63], which reported a standardized effect size for early TMS (up to 200 ms; *g =* 0.8) and for late TMS (after 200 ms; *g =* 0.5).

*Data filtering.* Participants with an overall accuracy in the experimental trials close to chance levels (less than 60% accuracy) in Experiments 1 and 2 were excluded from analyses and replaced. The data of such participants were not used during Bayesian sample updating nor for our main analyses. Additionally, we excluded and replaced participants in the case of technical or other difficulties, if data loss was greater than 20% of the total experimental trials. Further, the slowest and fastest responses were removed from the analyses. To do so, we filtered each participant's responses and excluded any data that concerned response times that were further than 3 standard deviations (s.d.) away from each participant's mean reaction time. Assuming that the reaction times of each participant are normally distributed, we expected less than 0.5% of the data of each participant to be excluded from the main analyses.

## 3. Results

### 3.1. Registered analyses

#### 3.1.1. Experiment 1

The 36 participants in Experiment 1 averaged 71.7% (s.d. = 6.1%) overall accuracy for the VSTM task. Filtering the reaction times that were 3 s.d. further away from each participant's mean reaction time excluded on average 3.5% (s.d. = 0.64%) of trials. Following the sensory visual cortex localization approach described previously (see *Sensory visual cortex stimulation* section), phosphene induction was successful for 25 participants, who had an average phosphene threshold of 66.2% (s.d. = 16.5%) out of the total (100%) stimulation power of the Magstim Super Rapid^2^ stimulator. For participants who failed to perceive phosphenes a fixed power set at 65% of the stimulator maximum output was used, with the coil placed approximately 2 cm above and 0.5 cm laterally, as previously discussed (see *Sensory visual cortex stimulation* section; see also [94]). The TMS coil was placed on the right hemisphere of 19 participants and on the left hemisphere of the remaining 17 participants.

**Figure 5.**
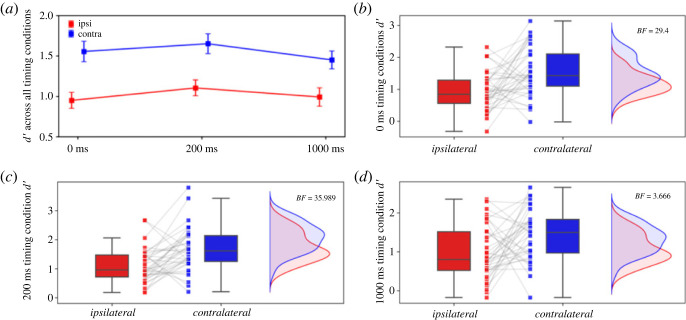
Preregistered analyses results for Experiment 1 with 36 participants. Detection sensitivity (*d′*) in Experiment 1 VSTM task performance across different TMS site coil placement and timing conditions. Because of monocular stimulus presentation, ipsilateral TMS (red) serves as the experimental condition, while contralateral TMS (blue) is the control condition. Mean *d′* is presented for (*a*) all timing conditions. Mean and individual *d′* scores are illustrated for (*b*) the 0 ms (outcome neutral), (*c*) the 200 ms (early VSTM maintenance) and (*d*) the 1000 ms (late VSTM maintenance) conditions. TMS: transcranial magnetic stimulation, VSTM: visual short-term memory.

Three hypotheses were preregistered for Experiment 1 (table 1), which aimed to investigate the role of the sensory visual cortex, by comparing the ipsilateral (experimental) to the contralateral (control) TMS site conditions, during three different VSTM phases, as follows: perceptual processing (H1), early visual information maintenance (H2) and late visual information maintenance (H3). The results of our registered analyses concerning Experiment 1 are summarized in table 2 and presented visually in figure 5.

**Table 2.** Data and analyses concerning the registered analyses of Experiment 1 (*n =* 36). TMS: transcranial magnetic stimulation.

hypothesis	TMS timing (ms)	TMS site	mean (s.d.) *d′*	Bayes factor (*BF*_10_)
H1	0	ipsilateral	0.95 (0.61)	29.40
		contralateral	1.56 (0.78)	
H2	200	ipsilateral	1.11 (0.59)	35.99
		contralateral	1.65 (0.75)	
H3	1000	ipsilateral	0.99 (0.69)	3.67
		contralateral	1.45 (0.67)	

For H1 the Bayesian paired *t*-test on mean *d′* regarding the outcome neutral (positive control) condition (0 ms TMS timing condition; figure 5*b*) revealed that the methods implemented in Experiment 1 were adequate to test for differences between the ipsilateral and contralateral conditions (*BF*_10_ = 29.40). This was reflected by a decrease in mean *d′* in the ipsilateral (mean *d′* = 0.95, s.d. = 0.61) compared with the contralateral (mean *d′* = 1.56, s.d. = 0.78) 0 ms TMS timing condition. This finding replicates the expected, consistent, inhibitory effect on VSTM performance due to sensory visual cortex TMS during visual perception processes (for reviews see [16,63]). Further, robustness analyses revealed that evidence, as reflected by the *BF*_10_, remains above the predefined threshold (*BF*_10_ > 3) for various prior distribution widths, including narrow (*r =* 0.3), wide (*r =* 1) and ultrawide (*r =* 1.5) scale widths (figure 3*a*). As such, these results are in line with our H1, where a difference between the ipsilateral and contralateral conditions was anticipated when sensory visual cortex TMS is induced at 0 ms, thus confirming the essential role of sensory visual cortex processes during perception.

Evidence for a similar inhibitory effect was found for H2 (figure 5*c*), which explored the early maintenance of visual information during VSTM that was reflected in the 200 ms TMS timing condition. In detail, the Bayesian paired *t*-test for the 200 ms TMS timing condition (early VSTM maintenance processes), indicated that mean *d′* was decreased in the ipsilateral (mean *d′* = 1.11, s.d. = 0.59) compared with the contralateral (mean *d′* = 1.65, s.d. = 0.75) condition (*BF*_10_ = 35.99). As indicated by the robustness analyses, the *BF*_10_ was consistently above the threshold (*BF*_10_ > 3) for narrow (*r =* 0.3), wide (*r =* 1) and ultrawide (*r =* 1.5) prior scale widths (figure 3*b*). Here, we hypothesized that evidence for a difference between the ipsilateral and contralateral conditions will be found when sensory visual cortex TMS is induced at 200 ms. Aligned with H2, the results indicated the presence of a difference, therefore confirmed the involvement of the sensory visual cortex during the early maintenance of visual information.

Analogous effects of reduced VSTM performance were found for H3 that concerned the late maintenance of visual information during VSTM (figure 5*d*); a condition that was echoed in the 1000 ms TMS timing condition. The Bayesian paired *t*-test for the 1000 ms TMS timing condition (late VSTM maintenance processes) revealed a decrease in mean *d′* when comparing the ipsilateral (mean *d′* = 0.99, s.d. = 0.69) with the contralateral (mean *d′* = 1.45, s.d. = 0.67) conditions (*BF*_10_ = 3.67). The robustness analyses showed that the threshold (*BF*_10_ > 3) was surpassed for a narrow prior (*r =* 0.3), but not for a wide (*r =* 1; *BF*_10_ = 2.59) and ultrawide (*r =* 1.5; *BF*_10_ = 1.9) prior (figure 3*c*). Despite not reaching the threshold for wider priors, the *BF*_10_ still indicates greater likelihood for the alternative hypothesis, thus favouring the presence of a difference, with an increasing trend as more participants are included. As with the previous hypotheses, the results for H3 were consistent with our hypothesis that evidence of a difference between the ipsilateral and contralateral conditions will be present when sensory visual cortex TMS is induced at 1000 ms. Hence, this evidence supports the involvement of the sensory visual cortex during the late maintenance of visual information during VSTM.

Taken together, these results support the involvement of the sensory visual cortex during VSTM. Further to the inhibitory effects found in the outcome neutral condition, which confirmed the reliability of our methods, the inhibitory TMS effects observed during the 200 and 1000 ms stimulation timing conditions, signify the involvement of the sensory visual cortex during early and late VSTM maintenance.

#### 3.1.2. Experiment 2

The average overall accuracy in the VSTM task of Experiment 2 of the 28 participants was 71.5% (s.d. = 6.6%). Reaction time filtering (excluding trials with reaction times 3 s.d. further from each participant's mean reaction time) resulted in the exclusion of 3.7% (s.d. = 0.86%) of trials, on average, for each participant. Phosphene induction was successful for 18 participants with an average phosphene threshold of 54.5% (s.d. = 21.8%). As in Experiment 1, a fixed output power set at 65% was used, with the coil placed approximately 2 cm above and 0.5 cm laterally, for participants who failed to perceive phosphenes (see also *Sensory visual cortex stimulation* section). For 15 participants, the coil was placed on the right hemisphere.

**Figure 6.**
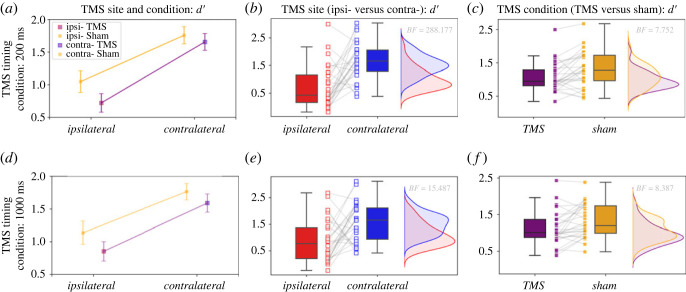
Preregistered analyses results for Experiment 2 with 28 participants. Detection sensitivity (*d′)* in Experiment 2 VSTM task performance across different TMS and sham conditions, different site coil placement and different timing conditions. For the 200 ms condition (top row) mean *d′* are shown for (*a*) both real (purple) and sham (orange) TMS, for the ipsilateral (red) and contralateral (blue) conditions. (*b*) Mean and individual *d′* scores between the ipsilateral and contralateral 200 ms conditions are shown only for the real TMS condition. (*c*) Mean and individual *d′* scores between sham and real TMS across hemispheres in the 200 ms condition. Results for the 1000 ms condition are illustrated in the bottom row. Mean *d′* for (*d*) real and sham TMS, for the ipsilateral and contralateral conditions. Mean and individual *d′* scores for the 1000 ms condition between (*e*) the ipsilateral and contralateral coil placement and between (*f*) real and sham stimulation. TMS: transcranial magnetic stimulation, VSTM: visual short-term memory.

Four hypotheses were preregistered for Experiment 2, which introduced sham stimulation. Two hypotheses concerned replicating the findings of Experiment 1 for early (H4) and late (H6) VSTM maintenance. To replicate the previous findings (H2 and H3 of Experiment 1), data from the sham TMS conditions were excluded from the analyses pertaining to H4 and H6. The remaining two hypotheses examined the role of the sensory visual cortex by comparing real with sham stimulation during early (H5) and late (H7) stimulation. The results of the registered analyses of Experiment 2 are summarized in table 3 and presented in figure 6.

**Table 3.** Data and analyses concerning the registered analyses of Experiment 2 (*n* = 28). TMS: transcranial magnetic stimulation.

hypothesis	TMS timing (ms)	TMS site	mean (s.d.) *d′*	Bayes factor (*BF*_10_)
H4	200	ipsilateral (TMS only)	0.72 (0.76)	288.18
		contralateral (TMS only)	1.66 (0.69)	
H5	1000	ipsilateral (TMS only)	0.85 (0.80)	15.49
		contralateral (TMS only)	1.59 (0.75)	
H6	200	TMS	1.08 (0.44)	7.75
		Sham	1.30 (0.54)	
H7	1000	TMS	1.11 (0.46)	8.39
		Sham	1.31 (0.45)	

The results related to H4 replicated our findings from Experiment 1 regarding the involvement of the sensory visual cortex during the early maintenance of visual information in VSTM (figure 6*b*). The Bayesian paired *t-*test showed that when real TMS is induced at 200 ms the ipsilateral (mean *d′* = 0.72, s.d. = 0.76) compared with the contralateral (mean *d′* = 1.65, s.d. = 0.69) mean *d′* is decreased (*BF*_10_ = 288.18). The *BF*_10_ remained consistently above the threshold (*BF*_10_ > 3) for narrow (*r =* 0.3), wide (*r =* 1) and ultrawide (*r =* 1.5) prior scale widths according to robustness analyses (figure 4*a*). This result replicates the findings of Experiment 1 (H2), and further strengthens our hypothesis that the sensory visual cortex is a necessary component of VSTM involved in the early maintenance of visual information.

Further, we analysed the differences between sham and real stimulation for the 200 ms TMS timing condition (H5; figure 6*c*). As previously described (see *Experimental design* and *Analysis plan* sections), this analysis was aimed to investigate the involvement of the sensory visual cortex in VSTM maintenance, in the case where the ipsilateral and contralateral comparisons were insufficient to do so (table 1), and to confirm the direction of the TMS effects (inhibitory versus facilitatory). These analyses were performed across the brain hemispheres, independent of the coil placement condition (i.e. without considering the ipsilateral or contralateral conditions). The results of the Bayesian paired *t-*test indicated that mean *d′* was reduced in the real TMS (mean *d′* = 1.08, s.d. = 0.44) compared with the sham TMS (mean *d′* = 1.3, s.d. = 0.54) condition (*BF*_10_ = 7.75), an effect that remained robust over the threshold (*BF*_10_ > 3) across a narrow (*r =* 0.3), wide (*r =* 1) and ultrawide (*r =* 1.5) prior (figure 4*b*). In addition to strengthening the evidence in favour of the involvement of the sensory visual cortex in early VSTM maintenance, this finding confirms the inhibitory effects of TMS induced at 200 ms, which were evident by the ipsilateral and contralateral comparisons (H2 and H4).

As for the late maintenance of visual information, we initially tested for differences between the ipsilateral and contralateral TMS conditions when stimulation was induced at 1000 ms (H6; figure 6*e*) and once again replicated the results of Experiment 1. Specifically, evidence for a difference was provided by the Bayesian paired *t*-test, showing decreased *d′* for the ipsilateral (mean *d′* = 0.85, s.d. = 0.8) compared with the contralateral (mean *d′* = 1.59, s.d. = 0.75) condition (*BF*_10_ = 10.84). Robustness analyses showed that the *BF*_10_ surpassed the threshold (*BF*_10_ > 3) for priors with narrow (*r =* 0.3), wide (*r =* 1) and ultrawide (*r =* 1.5) widths (figure 4*c*). As was the case with early maintenance, results for H6 replicated the effect that was found in Experiment 1 and provided additional evidence for the involvement of the sensory visual cortex during late VSTM information maintenance.

For the final registered hypothesis, H7, we investigated the 1000 ms TMS timing condition for differences between sham and real stimulation (figure 6*f*). As indicated by the Bayesian paired *t*-test, mean *d′* in the real TMS condition (mean *d′* = 1.11 s.d. = 0.46) was decreased in comparison with the sham TMS (mean *d′* = 1.34, s.d. = 0.45) condition (*BF*_10_ = 8.39). The *BF*_10_ threshold (*BF*_10_ > 3), was exceeded for narrow (*r =* 0.3), wide (*r =* 1) and ultrawide (*r =* 1.5) prior widths, as reflected by robustness analyses (figure 4*d*). This finding offers additional evidence for the involvement of the sensory visual cortex in late VSTM maintenance and provides further support for the inhibitory TMS effects, as was the case with the ipsilateral and contralateral comparisons for stimulation induced at 1000 ms (H3 and H6).

In general, the results from Experiment 2 replicate the findings of Experiment 1 and strengthened the evidence in favour of the involvement of the sensory visual cortex during VSTM maintenance. The introduction of a sham condition upheld the inhibitory TMS effects that were also found in the ipsilateral versus the contralateral comparisons, which indicates that sensory visual cortex TMS during the VSTM maintenance period impairs VSTM performance. Overall, since TMS disrupted early and late VSTM maintenance processes, our findings are aligned with the sensory recruitment hypothesis. In the following section, we discuss some exploratory analyses, which were not registered during Stage 1 of this registered report.

### 3.2. Exploratory analyses

Exploratory analyses were performed using the JASP statistical software package ([95], v. 0.16.3; https://jasp-stats.org). Of note, to compute Bayesian analyses, Jamovi uses a JASP-based package, thus we anticipate that results are consistent between the two statistical packages. The exploratory repeated measures analysis of variance (rmANOVA) tests were informed using the priors suggested by Rouder *et al*. [96], which assume Cauchy distributions centred on 0 (fixed effects *r* = 0.5; random effects *r* = 1). For the exploratory *t-*test, we used a Cauchy distribution centred on 0 with a width set to 0.58, based on the overall TMS effect that was found in past meta-analytic work [63].

#### 3.2.1. Experiment 1

Previous studies have reported different TMS effects across stimulation sites for the different stimulation timing conditions [60,62,64]. To explore these effects for our Experiment 1, we conducted a Bayesian rmANOVA for the TMS site and TMS timing conditions. This created a two (ipsilateral versus contralateral) by three (0, 200, 1000 ms) model (figure 5*a*). To explore the model that better represents the data, we conducted analysis on the factor effects by calculating the likelihood ratio representing the change from prior odds to posterior odds for each factor in the model averaged by all the models that include each factor (*BF*_incl_). The *BF*_incl_ for all factors and interactions are provided in table 4. In detail, the inclusion of the TMS site factor resulted in the highest *BF*_incl_ (*BF*_incl_ = 23.01). Also, there was moderate evidence against the inclusion of an interaction of TMS site and TMS timing (*BF*_incl_ = 0.34); however, the inclusion of the TMS timing factor resulted in indecisive evidence (*BF*_incl_ = 0.73). The results of the exploratory Bayesian rmANOVA inform us that, in line with the registered analyses of Experiment 1, a TMS site (ipsilateral versus contralateral) effect is evident and that an interaction with TMS timing is unlikely.

**Table 4.** Experiment 1 (*n* = 36) Bayesian repeated measures ANOVA analysis of effects. *BF*_incl_ is calculated as the likelihood ratio representing the change from prior odds to posterior odds for each factor in the model averaged by all the models that include each factor. TMS: transcranial magnetic stimulation.

model	Bayes factor (*BF*_incl_)
TMS site	23.01
TMS time	0.73
TMS site × TMS time	0.34

#### 3.2.2. Experiment 2

A similar exploratory Bayesian rmANOVA, was implemented to explore the possible effects across the TMS condition, site and timing factors. In detail, we explored a two (real versus sham) by two (ipsilateral versus contralateral) by two (200, 1000 ms) model (figures 6*a*,*d*). As with Experiment 1, we performed an analysis of effects by calculating a *BF*_incl_ for each factor and interaction included in the model. The *BF*_incl_ resulting from this analysis are presented in table 5. Specifically, the highest *BF*_incl_ was produced by the TMS condition model (*BF*_incl_ = 31.45), followed by that of the TMS site model (*BF*_incl_ = 15.45). The models including solely TMS timing, or TMS timing interactions resulted in low *BF*_incl_ (all *BF*_incl_ < 0.37; table 5 for details), thus providing moderate to strong evidence against any timing effects or interactions. The results of the Bayesian rmANOVA are analogous to those registered for Experiment 2, where both a TMS condition (sham versus real) and TMS site (ipsilateral versus contralateral) effects were found, but differences across timings are unlikely.

**Table 5.** Experiment 2 (*n* = 28) Bayesian repeated measures ANOVA analysis of effects. *BF*_incl_ is calculated as the likelihood ratio representing the change from prior odds to posterior odds for each factor in the model averaged by all the models that include each factor. TMS: transcranial magnetic stimulation.

model	Bayes factor (*BF*_incl_)
TMS condition	31.45
TMS site	15.45
TMS condition × TMS site	1.73
TMS time	0.21
TMS condition × TMS time	0.18
TMS site × TMS time	0.36
TMS condition × TMS site × TMS time	0.08

To further explore the effects of the TMS condition and the TMS site factors, we performed *post hoc* Bayesian paired *t*-tests. Evidence for an overall real compared with sham TMS was found (*BF*_10_ = 60.5), signifying impaired performance in the real TMS (mean *d′* = 1.09, s.d. = 0.42) compared with the sham TMS (mean *d′* = 1.29, s.d. = 0.91) condition. This was an expected finding considering the inhibitory effects that were confirmed through our registered analyses. Further, an overall ipsilateral versus contralateral difference was shown (*BF*_10_ = 41.85), indicating that overall performance in the ipsilateral condition (mean *d′* = 0.91, s.d. = 0.76) was worse compared with the contralateral condition (mean *d′* = 1.65, s.d. = 0.62). This overall TMS site (ipsilateral versus contralateral) effect is probably attributed to the consistent stimulation of one brain hemisphere, which can lead to perceptual inhibition [16,97]. A deeper investigation into these factors showed that the real versus sham effects are evident only in the ipsilateral conditions in both the 200 ms (ipsilateral TMS mean *d′* = 0.72, s.d. = 0.76; ipsilateral sham mean *d′* = 1.05, s.d. = 0.89; *BF*_10_ = 6.67) and the 1000 ms (ipsilateral TMS mean *d′* = 0.85, s.d. = 0.8; ipsilateral sham mean *d′* = 1.14, s.d. = 0.95; *BF*_10_ = 3.02) timing conditions. Real versus sham TMS comparisons in the contralateral condition remained inconclusive for the 200 ms (contralateral TMS mean *d′* = 1.66, s.d. = 0.69; contralateral sham mean *d′* = 1.76, s.d. = 0.71; *BF*_10_ = 0.4) and 1000 ms (contralateral TMS mean *d′* = 1.59, s.d. = 0.75; contralateral sham mean *d′* = 1.76, s.d. = 0.68; *BF*_10_ = 1.56) timing conditions, since the *BF*_10_ failed to reach our predefined threshold (1/3 < *BF*_10_ < 3).

Overall, the exploratory analyses echo the results of our registered hypotheses, showing the inhibitory effects of TMS for both comparisons considering either the stimulation site (ipsilateral versus contralateral coil placement) or the stimulation condition (real or sham coil). Moreover, the analyses for both Experiment 1 and Experiment 2 revealed that no timing differences are evident, a finding that aligns with the results of our recent meta-analysis that systematically identified and analysed previous VSTM TMS studies [63].

## 4. Discussion

The aim of this preregistered study was to investigate if sensory visual cortex is a necessary component of the brain network involved in the short-term maintenance or storage of visual information. For this reason, our experimental methods were designed for overcoming methodological issues that were identified in previous TMS studies investigating similar questions. Overcoming these oversights required the monocular presentation of stimuli, and the use of stimuli comprised elemental visual features, such as orientation. In two experiments, we showed that TMS impairs VSTM task performance when induced during both early (200 ms) and late (1000 ms) visual information maintenance. The reliability of our methods, which were preregistered prior to any data collection, were confirmed by similar inhibitory TMS effects found during perception (outcome neutral condition; Experiment 1) and by sham TMS performance comparisons (Experiment 2). These results provide causal evidence for the involvement of the sensory visual cortex in VSTM maintenance, in line with the sensory recruitment hypothesis.

In Experiment 1, we showed that by ensuring monocular processing of orientation stimuli presented within 15° of visual angle, sensory visual cortex TMS on the ipsilateral—to the eye processing the information—brain hemisphere, resulted in impaired performance in an orientation VSTM task, compared with performance in the contralateral (control) condition (cf. [24,58,60,62]). Specifically, both early (200 ms) and late (1000 ms) sensory visual cortex TMS that was induced during the task's 2 s maintenance period impaired VSTM task performance. The same effect was replicated in Experiment 2. An analogous effect was evident for TMS induced simultaneously with stimulus presentation, during VSTM perceptual processes, parallel to the established role of the sensory visual cortex during visual perception [15–19,29], which confirmed the reliability of the early and late maintenance comparison findings. These results support the view of visual information storage within the sensory visual cortex, as proposed by sensory recruitment [20,23].

Further to replicating the results found in Experiment 1, Experiment 2 provided additional insight for the involvement of the sensory visual cortex in VSTM maintenance, by introducing sham TMS. Comparisons between real and sham stimulation revealed impaired VSTM performance, which was caused by real TMS (compared with VSTM performance in the sham TMS condition). The importance of this finding is twofold. Firstly, the use of the sham coil introduces a second control condition, further to the contralateral control condition. This addition is pivotal, since it has been previously suggested that multiple control conditions need to be considered so that TMS inferences can be limited within a specific brain network [57,98,99]. Therefore, the findings from the real versus sham stimulation comparisons from Experiment 2, reverberated the evidence in favour of sensory recruitment that was evident between the stimulation site (ipsilateral versus contralateral) comparisons.

Second, to correctly interpret the direction of the TMS effects found between the ipsilateral compared with the contralateral comparisons, the sham TMS condition is essential. Previous TMS studies presented contradictory results, with some showing inhibitory TMS effects [59–62,64] and others supporting facilitatory TMS effects [58,88,100]. Moreover, our recent meta-analysis of these studies was unable to distinguish between the direction of effects, because of the various methods employed in each experiment and due to the failure to account for monocular stimuli presentation [63]. For example, because of the neural basis [65–67] and the feedforward and feedback mechanisms ([45,85]; see also [84]) of the sensory visual cortex, it is possible for visual information to enter either or both brain hemispheres. Thus, the direction of effect (facilitation versus inhibition) caused by sensory visual cortex TMS is unavoidably biased by the definition of the experimental and control conditions. As an example, if the ipsilateral site is defined as the experimental condition, then impaired performance will be interpreted as inhibition, but might in fact reflect facilitation TMS effects of the contralateral condition. This insight related to the direction of effect, by the introduction of sham TMS, helps clarify the mixed results reported in the literature and shows that the observed results were indeed due to inhibitory TMS effects.

Nevertheless, facilitation TMS effects during VSTM maintenance cannot be completely ruled out. This is attributed to the possibility of different maintenance processes employed by VSTM, such as activity-silent (or latent) memory representations [101], and the different TMS effects on such processes [102]. Specifically, previous work has discussed that the direction of TMS effects, whether inhibitory or facilitatory, depend on the attentional state of the recalled item [103]. For example, it has been shown that sensory visual cortex TMS causes inhibitory effects for attended memory items [100,104] and facilitatory effects for unattended items ([100]; although this finding was not replicated in [104]). In turn, recent evidence has suggested that the attentional state can lead to different VSTM storage processes, where attended stimuli are maintained through sustained neural activations, whereas unattended items are maintained through activity-silent mechanisms (e.g. synaptic weight changes; [105]; for a review see [29]). The two experiments carried out here, even though they consistently revealed inhibitory TMS effects, are limited to testing the effects solely on attended—behaviourally relevant—items. However, it is possible that TMS during the maintenance of unattended items leads to opposite, facilitatory, effects [100,102], but such effects remain unexplored because of the behavioural relevance of all stimuli in our task. Along these lines, future work should study this possibility, by combining the monocularly presented VSTM task, with double retrospective cueing (e.g. [106]), which will enable the manipulation of attention between behaviourally relevant (sustained-activity) and irrelevant (activity-silent) memory items.

Previous work has presented mixed results regarding the TMS timing effects, with some studies indicating stronger TMS effects for earlier stimulation (up to 200 ms; [60,62]), compared with later stimulation (400 ms, [64]; 900 ms, [60]); however, other studies indicated that TMS at 200 ms was stronger [64]. In our study, exploratory analyses of data from Experiment 1 provided moderate evidence against an interaction between the site and the timing of the stimulation for the effects of TMS, while Experiment 2 provided moderate to strong evidence against any timing effects. Despite their exploratory nature, these results are aligned with a recent meta-analysis establishing that TMS effects are similar between earlier and later stimulation [63]. Notably, a recent review [12] argued that the stronger effects for earlier TMS found in some previous studies [60,62] can be taken as evidence against the storage of information by the sensory visual cortex during VSTM. This argument was further complemented by the null finding in the study of van de Ven *et al*. [64] for TMS at 400 ms. However, we argue that a weaker effect during later stimulation does not correspond to the absence of an effect. On the contrary, as reflected by our results, even though the likelihood of the evidence is lower for later stimulation, the effects of TMS cannot be differentiated based on timing of the stimulation (see also [63]). Along these lines and in contrast to the argument of Xu [12], we propose that, taken together, evidence from TMS supports the idea that sensory visual cortex is an essential part of the network involved in VSTM.

A possible explanation of the different results concerning stimulation timings in previous TMS studies is probably attributed to the processing of information by both sensory visual cortex hemispheres (for a similar argument see [63]). Because stimuli in previous TMS work were presented binocularly, it is possible that information was processed by both the ipsilateral and contralateral sensory visual cortex [46,66,67]. Since both hemispheres are employed for short-term maintenance, it is likely that feedforward and feedback mechanisms are used for maintenance (e.g. [45]; for a review see [51]), which can improve VSTM representation fidelity given a longer maintenance period ([46,107]; see also [51]). In the current study, because stimuli were restricted to enter only one sensory visual cortex hemisphere, feedforward and feedback processes by binocular sensory visual cortex neurons [46,74] were less likely to be engaged to protect representations given the additional maintenance time. Hence, representations remained protected solely by the ipsilateral brain hemisphere, which was then susceptible to the detrimental TMS effects.

Another potential contributing factor to the lack of a TMS difference between early (200 ms) versus late maintenance (1000 ms) could be the memory load used in this study. Our current findings are consistent with previous research on young adults, which found that when the memory load was low, there was no difference in VSTM performance at 200, 1000 and even 1800 ms [108]. However, when the memory load was high and exceeded VSTM capacity limits, earlier VSTM maintenance (200 ms) was associated with better performance compared with later maintenance (1000 ms) [108]. Van de Ven *et al.* [64] also found that TMS effects on the sensory visual cortex were only present when participants were required to maintain a high memory load in VSTM, but not during low memory load. This suggests that VSTM load may influence sensory visual cortex activity, a conclusion supported by psychophysical [109–111] and brain imaging evidence [33,112]. These findings may explain the different TMS effects seen in previous studies (see also [63]). Future research could modify the monocular VSTM task used in this study and examine the effects of TMS on various VSTM memory load conditions at different stimulation timing points.

Despite preregistration, this study is still subject to limitations. One such limitation relates to the limited information provided in terms of the nature of VSTM performance. For example, to understand VSTM, some researchers prefer continuous report tasks over change-detection tasks (see [113]). In these tasks, mixture (probabilistic) models are fitted on a recalled continuous feature (e.g. colour or orientation) that provide sensitive measures for two [80], three [114] or more components [115], and can provide greater insight regarding the underpinnings of VSTM (e.g. guess proportion, memory precision, absolute error). However, such models require a lot of trials to reach sufficient power, which makes TMS studies difficult to be carried out on a large sample size (e.g. [60]). Here, to have an adequate sample size for our registered analyses (see [116]), we opted for the use of a change-detection task, which was deemed as an appropriate approach (e.g. [117]) to understand our main research question of whether the sensory visual cortex contributes to VSTM maintenance. Notably, according to a recent review of the TMS literature [63], in our two experiments, we recruited the largest sample size to date (*n* = 36 in Experiment 1; *n* = 28 in Experiment 2), compared with a range between eight [60] and 21 [62] participants in previous studies investigating sensory recruitment. Given that, with our large sample size, the involvement of the sensory visual cortex in VSTM maintenance has been supported by our two experiments, future TMS work can adjust our task to fit mixture models for monocularly presented continuous orientation report tasks, to extend the question from if to how the sensory visual cortex contributes to VSTM storage.

Lastly, despite the benefits of the sample updating with stopping rule design [118], this approach can also be subject to limitations. In our study, the stopping rule was focused on our registered analyses, which enabled us to tailor our sample size accordingly, so that we gathered adequate evidence regarding our hypotheses (table 1), as reflected by the *BF*, while preserving resources [118–121]. However, the sole focus on the registered analyses and their proposed prior distributions (table 1), resulted in some inconclusive results in the exploratory and robustness analyses, since the predefined threshold was not reached (1/3 < *BF*_10_ < 3). Even though these exploratory analyses were not the focus to drive the theory of the two experiments presented here, it is possible that with additional participants, the proposed *BF*_10_ threshold for additional analyses would have been reached (e.g. figure 3*c*). As such, future studies using Bayesian designs could rely on different approaches for sample size determination, such as simulations that can inform the minimum required number of participants for various study designs [116,120,122]. Alternatively, future work could set two different *BF* thresholds for the stopping rule and the decision rule,^2^ to allow for additional sensitivity in case exploratory analyses are anticipated. For example, the decision rule to support a hypothesis might be set to a specific *BF* threshold (e.g. 1/3 < *BF*_10_ < 3), but the stopping rule is set at a higher threshold (e.g. 1/6 < *BF*_10_ < 6).

Conclusively, in two experiments, we provide causal evidence for the involvement of the sensory visual cortex in VSTM maintenance through TMS. Between hemisphere comparisons revealed inhibitory TMS effects, as reflected by impaired VSTM task performance in the stimulated sensory visual cortex hemisphere during perceptual, early maintenance and late maintenance VSTM processes. These effects reverberated in comparisons between sham and real TMS conditions during both early and late VSTM maintenance. Overall, these effects support the sensory recruitment hypothesis, which proposes shared neural underpinnings within the sensory visual cortex for the perception and storage of elemental visual features during VSTM.

Stage 1 PCI-RR recommendation & reviews: https://rr.peercommunityin.org/articles/rec?id=142.

Stage 2 PCI-RR recommendation & reviews: https://rr.peercommunityin.org/articles/rec?id=362.

This Registered Report was submitted to Royal Society Open Science following peer review and recommendation for Stage 2 acceptance at the Peer Community In (PCI) Registered Reports platform. Full details of the peer review and recommendation of the paper are available at PCI.

After submission to the journal, the paper received no additional external peer review, but was accepted on the basis of the Editor's recommendation according to our PCI Registered Reports policy https://royalsocietypublishing.org/rsos/registered-reports#PCIRR.

## Data Availability

All data, code and materials used in this study are available at https://osf.io/d9bqk/?view_only=e3d4215a8cd6411e9256d259197bb875. The data are provided in electronic supplementary material [123].

## Footnotes

1 The number of trials in Experiments 1 and 2 have been increased compared with the registered trial numbers, due to correcting a mistake in the calculation of the required trials (432 instead of 360 that were registered in Experiment 1 and 576 instead of 512 that were registered in Experiment 2). This error was corrected prior to any data collection and after receiving the recommender's approval on 22 June 2022.

2 We thank Prof. Zoltan Dienes, the recommender of this registered report, for proposing this possible solution to the issue of sample updating with stopping rules.
