# Supplementary material for: Causal evidence for the role of the sensory visual cortex in visual short-term memory maintenance
Source: R Soc Open Sci. 2023 Apr 19;10(4):230321. doi: 10.1098/rsos.230321 (PMC10113812; doi:10.1098/rsos.230321)
Supplement: Supplementary file 1 [file rsos230321_review_history.pdf]

THE ROYAL SOCIETY
PUBLISHING

ROYAL SOCIETY
OPEN SCIENCE

Causal evidence for the role of the sensory visual cortex in visual short-term memory maintenance

Phivos Phylactou, Andria Shimi and Nikos Konstantinou

Article citation details
R. Soc. open sci. **10**: 230321.
http://dx.doi.org/10.1098/rsos.230321

Review timeline

Original submission:	21 March 2023
Revised submission:	22 March 2023
Final acceptance:	27 March 2023

Note: Reports are unedited and appear as submitted by the referee. The review history appears in chronological order.

Review History

Decision letter (RSOS-230321.R0)

Dear Mr Phylactou

On behalf of the Editor, I am pleased to inform you that your Manuscript RSOS-230321 entitled "Causal evidence for the role of the sensory visual cortex in visual short-term memory maintenance" has been accepted in principle for publication in Royal Society Open Science. The reviewers' and editors' comments are included at the end of this email.

Please note there is no action required from you at this stage - we will now submit the Stage 2 report on your behalf.

Kind regards,
Royal Society Open Science Editorial Office
Royal Society Open Science
openscience@royalsociety.org

on behalf of Professor Chris Chambers (Associate Editor) and Chris Chambers (Registered Reports Editor, Royal Society Open Science)
openscience@royalsociety.org

Reports © 2023 The Reviewers; Decision Letters © 2023 The Reviewers and Editors; Responses © 2023 The Reviewers, Editors and Authors. Published by the Royal Society under the terms of the Creative Commons Attribution License http://creativecommons.org/licenses/by/4.0/, which permits unrestricted use, provided the original author and source are credited

Associate Editor Comments to Author (Professor Chris Chambers):
Associate Editor: 1
Comments to the Author:
(There are no comments.)

Associate Editor: 2
Comments to the Author:
(There are no comments.)

Author's Response to Decision Letter for (RSOS-230321.R0)

See Appendix A.

Decision letter (RSOS-230321.R1)

Dear Mr Phylactou:

I am pleased to inform you that your manuscript entitled "Causal evidence for the role of the sensory visual cortex in visual short-term memory maintenance" is now accepted for publication in Royal Society Open Science.

If you have not already done so, please ensure that you send to the editorial office an editable version of your accepted manuscript, and individual files for each figure and table included in your manuscript. You can send these in a zip folder if more convenient. Failure to provide these files may delay the processing of your proof.

Please remember to make any data sets or code libraries 'live' prior to publication, and update any links as needed when you receive a proof to check - for instance, from a private 'for review' URL to a publicly accessible 'for publication' URL. It is also good practice to add data sets, code and other digital materials to your reference list.

Royal Society Open Science is a fully open access journal. A payment may be due before your article is published. Please note that, if the corresponding author of your paper is based at an institution covered by one of our Transformative Agreement deals, your fees may be covered by the deal – please check the list of eligible institutions at https://royalsociety.org/journals/authors/read-and-publish/read-publish-agreements/. The Royal Society has partnered with Copyright Clearance Center's (CCC's) RightsLink service to allow authors to pay article processing charges or page charges. After your manuscript has been accepted, the corresponding author will receive an email from CCC with the subject "Please submit your article processing/open access charge(s)/page charges" inviting you to pay your charges or request an invoice. The email from CCC will come from the email domain @copyright.com (if you have any queries regarding fees, please see https://royalsocietypublishing.org/rsos/charges or contact authorfees@royalsociety.org). If you request an invoice, it will be sent to you from CCC. It is important to be cautious about payment scams. If you receive an email or text message requesting payment and have any concerns, we recommend contacting us through our website, rather than clicking on any links. The Royal Society will never ask you to make a direct payment.

The proof of your paper will be available for review using the Royal Society online proofing system and you will receive details of how to access this in the near future from our production

office (openscience_proofs@royalsociety.org). We aim to maintain rapid times to publication after acceptance of your manuscript and we would ask you to please contact both the production office and editorial office if you are likely to be away from e-mail contact to minimise delays to publication. If you are going to be away, please nominate a co-author (if available) to manage the proofing process, and ensure they are copied into your email to the journal.

Please see the Royal Society Publishing guidance on how you may share your accepted author manuscript at https://royalsociety.org/journals/ethics-policies/media-embargo/. After publication, some additional ways to effectively promote your article can also be found here https://royalsociety.org/blog/2020/07/promoting-your-latest-paper-and-tracking-your-results/.

Your feedback matters - please spend 5 minutes leaving anonymous feedback about your experience of Registered Reports at this journal, as an author or reviewer: https://registeredreports.cardiff.ac.uk/feedback/feedback/decision_letter.php

This feedback is collected by the Registered Reports Community Feedback website, which is an independent service and research project, being undertaken by Cardiff University.

On behalf of the Editors of Royal Society Open Science, thank you for your support of the journal and we look forward to your continued contributions to Royal Society Open Science.

Kind regards,
Royal Society Open Science Editorial Office
Royal Society Open Science
openscience@royalsociety.org

on behalf of Professor Chris Chambers (Subject Editor).

Follow Royal Society Publishing on Twitter: @RSocPublishing
Follow Royal Society Publishing on Facebook:
https://www.facebook.com/RoyalSocietyPublishing/
Read Royal Society Publishing's blog:
https://royalsociety.org/blog/blogsearchpage/?category=Publishing

Appendix A

Causal evidence for the role of the sensory visual cortex in visual short-term memory maintenance

Phivos Phylactou, Andria Shimi, Nikos Konstantinou

Stage 1 PCI-RR recommendation & reviews:
https://rr.peercommunityin.org/user/recommendations?articleId=142&_signature=c134d0355cdf8d1ea9ae2576b80a589c9b10ade3

Stage 2 PCI-RR recommendation & reviews:
https://rr.peercommunityin.org/user/recommendations?articleId=362&_signature=4054c175223456de54a2b873806864cbdca63087

This Registered Report was submitted to Royal Society Open Science following peer review and recommendation for Stage 2 acceptance at the Peer Community In (PCI) Registered Reports platform. Full details of the peer review and recommendation of the paper at PCI Registered Reports may be found at the links below.

After submission to the journal, the paper received no additional external peer review, but was accepted on the basis of the Editor's recommendation according to our PCI Registered Reports policy https://royalsocietypublishing.org/rsos/registered-reports#PCIRR.
